# NARRATIVES OF WARTIME VOLUNTEERING AMONG OLDER ADULTS IN ISRAEL

**DOI:** 10.1093/geroni/igae098.3870

**Published:** 2024-12-31

**Authors:** Peter Sun, Liat Ayalon

**Affiliations:** Bar-Ilan University, Ramat Gan, Tel Aviv, Israel; Bar Ilan University, Bar Ilan University, HaMerkaz, Israel

## Abstract

Volunteering is an important vehicle for the health promotion of older adults, yet research on its role during times of war remain limited. This research aimed to explore the representation and rhetoric of older adult volunteers in the Israeli media during Operation Swords of Iron. Using the LexisNexis database, four Israeli newspapers were searched for English-language articles published between October 7, 2023 and August 7, 2024: Haaretz, The Times of Israel, Yediot Achronot, and The Jerusalem Post. After removing duplicates and articles that were not relevant to older adult volunteering, a total of 23 articles were retained for thematic analysis. To probe the sentiments of these narratives, sentences containing keywords about older adult volunteers were compared against a preexisting lexicon of semantic orientations. Thematic analysis revealed three themes: (1) Older adult volunteers as heroic and self-sacrificial amid the risks of war, (2) the virtue of being productive versus unproductive, and (3) older adults as agile first responders in situations of distress. Sentiment analysis further uncovered both positive and negative feelings that were collocated with the rhetoric on volunteering, such as the juxtaposition of older volunteers with the lack of supervision or training. The rhetoric of heroism, sacrifice, and productivity suggest the need to attend to age-related stereotypes of older adults. This research not only has implications for the health of older adults, but also for supporting older adult volunteers contributing towards large-scale public health challenges.

